# Sublingual clear cell carcinoma with liver metastasis: A case report and literature review

**DOI:** 10.1002/kjm2.12567

**Published:** 2022-06-15

**Authors:** Chia‐Chuan Chen, Po‐Hsuan Wu, Shih‐Chang Chuang, Hsuan‐Ying Huang

**Affiliations:** ^1^ Department of Surgery Kaohsiung Medical University Hospital Kaohsiung Taiwan; ^2^ Kaohsiung Medical University Kaohsiung Taiwan; ^3^ Department of Pathology Chang Gung Memorial Hospital Kaohsiung Branch Kaohsiung Taiwan

## 1 INTRODUCTION

Clear cell carcinoma, hyalinizing clear cell carcinoma (HCCC), and not otherwise specified (NOS) are rare, uncommon tumors. The most common sites of clear cell carcinoma are in the oral cavity and the major salivary gland is the second most common location. In the major salivary gland, there is less sublingual clear cell carcinoma and the overall outcomes are generally better in those cases. However, local recurrences and distant metastasis were reported in some cases, such as lung and bone.^1^ No case of liver metastasis was previously reported except for one autopsy case in the current study.^2^ We report a sublingual gland clear cell carcinoma case without local recurrence but with distant liver metastasis.

## 2 CASE PRESENTATION

**FIGURE 1:**
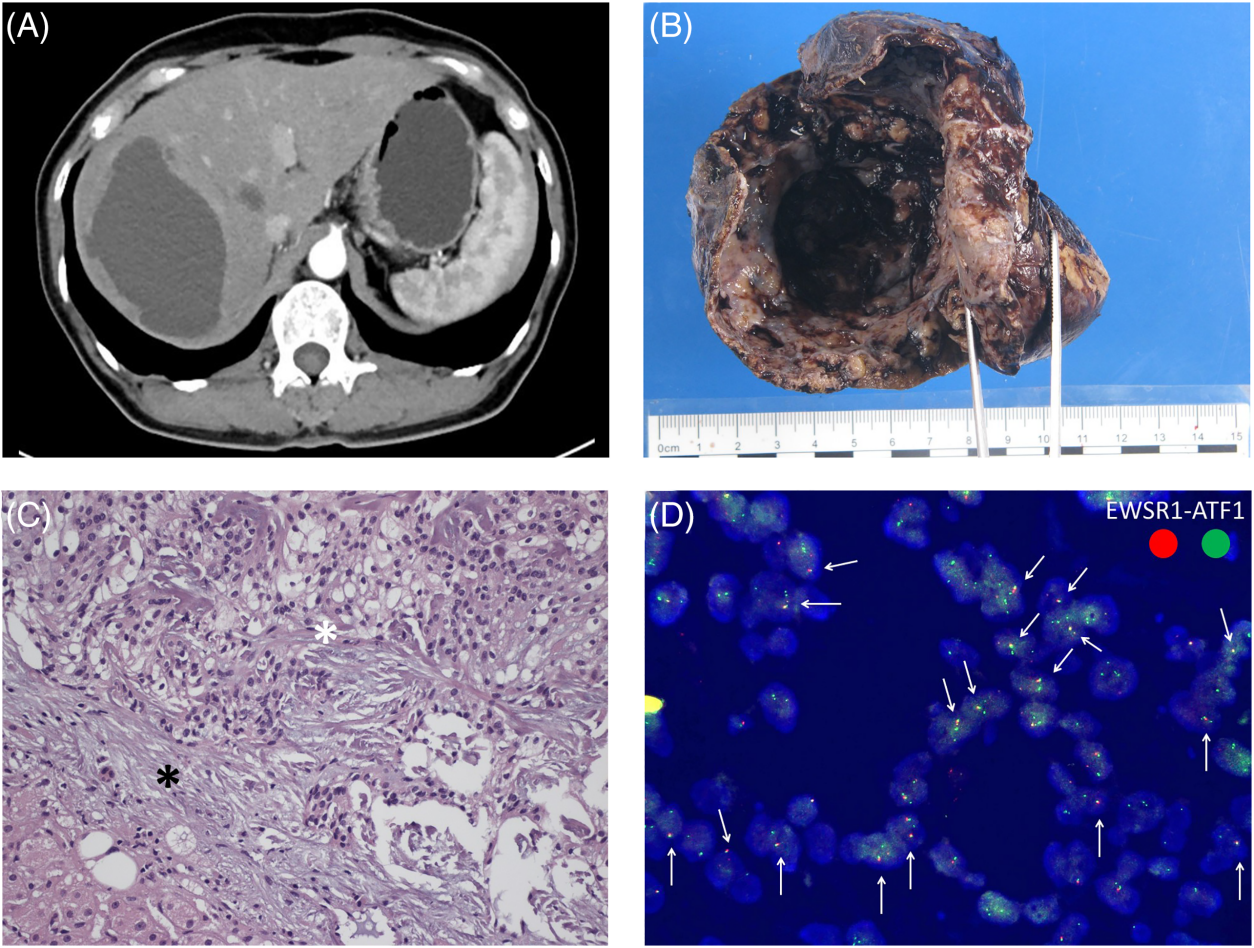
(A) Cystic lesion with capsule in segment 7 of the liver; (B) Liver specimen consists of brownish fluid accumulation with one huge cystic lesion; (C) Liver hyalinized clear cell carcinoma comprises clear cell part (white asterisk) and hyalinized stroma part (black asterisk). The clear cell part showed thick sheets of round to spindle clear neoplastic cells (white arrows) exhibiting normochromatic nuclei and clear to eosinophilic cytoplasm. The hyalinized stroma part showed desmoplastic stroma that is hyalinized; (D) Molecular marker EWSR1(red) and ATF1(green) were both positive, as arrows showed fluorescence microscopically.

A 56‐year‐old female presented with a right sublingual gland clear cell carcinoma was treated with wide excision in 2013. She underwent radiotherapy after the operation. There was no recurrence after radiotherapy. In 2019, the patient presented with painless abdominal fullness and was diagnosed with a 6‐cm cyst with wall thickening in segment 7 of the liver via abdominal sonography. Computed tomography of the abdomen revealed a rim‐enhanced lesion in segment 7 of the liver suspicious of abscess and distant metastasis. An echo‐guided fine needle biopsy showed the proliferation of benign glandular cells. Computed tomography of the oral cavity was negative for tumor recurrence or lymphadenopathy. However, after one and a half years of follow‐up, computed tomography of the abdomen disclosed enlargement of the cystic lesion (10 × 7 × 6 cm) with a thickened capsule in segment 7 of the liver (Figure 1A) and the patient underwent liver resection of segment 7. The specimen (Figure 1B) had brownish color fluid accumulation. It was a brownish and soft cystic lesion measuring 10.2 × 7.5 × 5.8 cm in size. The microscopic appearance (Figure 1C) showed clear cell neoplasms composed of thick sheets of round to spindle clear neoplasm cells exhibiting normochromatic nuclei and clear to eosinophilic cytoplasm. Pathology further confirmed metastasis from sublingual gland clear cell carcinoma with the molecular markers ESWR1(+) and ATF1(+) (Figure 1D).^3^ The patient was discharged 10 days after surgery and had been undergoing adjuvant chemotherapy for 1 year without recurrence.

## 3 DISCUSSION

Hyalinizing clear cell carcinoma is a painless lesion and rare low‐grade malignancy. It has a predilection for females over males (2:1) and the incidence increases with age, especially amongst 60–70 years old. The most common sites are the oral cavity, major salivary gland, and head/neck region. The oral cavity, including the tongue, palate, and floor of the mouth is mostly diagnosed with HCCC. Major salivary gland cases occur in the parotid, submandibular, and sublingual regions. HCCC represented <1% in all malignant salivary gland tumors. Head and neck are also reported in some cases. Some cases may present with distant metastasis. Solar et al. reviewed HCCC case reports from 1983 to 2007, and 2 out of 44 cases had lung distant metastasis cases.^3^ Yamashita et al. reported a patient with multiple metastases on autopsy, including lung, pleura, bone, brain, lung, liver, gallbladder, pancreas, omentum, and skin metastases.^2^

It is critical to distinguish HCCC from more aggressive cancers ensuring proper treatment and management. The diagnosis of clear cell carcinoma is based on clinical, histology, immunohistochemical, and genetic features. A review paper stated that clear cell carcinoma was common in perineural invasion and mucosal involvement.^3^ Histologically, HCCC presented proliferating epithelial cells with clear cytoplasm. Some had thin cords and small nests, focal cribriform patterns, dual hyalinizing, and fibrocellular storms. Mucin was commonly seen in single cells. Immunohistochemically, HCCC is positive for p62, CK5, CK7, CK14, CK19, and HMWK and negative for S‐100, SMA, PAX8, and PAS with distaste, actin, and calponin. The molecular marker EWSR1‐ATF1 was specific to diagnose HCCC(3).

There is a lack of comprehensive understanding of its clinical characteristics and treatment plan. Most cases were treated with surgery and radiotherapy for primary cancer without local or distant metastasis.^4^ Postoperation radiotherapy was used in patients with local recurrence.^5^Some cases with distant metastasis received chemotherapy.^1^, ^4^ The chemotherapy regimen was not discussed in the literature. Careful observation and close follow‐up of these patients should be warranted.

## CONFLICT OF INTEREST

All authors declare no conflict of interest.
